# Bacterial and *Pneumocystis* Infections in the Lungs of Gene-Knockout Rabbits with Severe Combined Immunodeficiency

**DOI:** 10.3389/fimmu.2018.00429

**Published:** 2018-03-09

**Authors:** Jun Song, Guoshun Wang, Mark J. Hoenerhoff, Jinxue Ruan, Dongshan Yang, Jifeng Zhang, Jibing Yang, Patrick A. Lester, Robert Sigler, Michael Bradley, Samantha Eckley, Kelsey Cornelius, Kong Chen, Jay K. Kolls, Li Peng, Liang Ma, Yuqing Eugene Chen, Fei Sun, Jie Xu

**Affiliations:** ^1^Center for Advanced Models for Translational Sciences and Therapeutics, University of Michigan Medical Center, University of Michigan Medical School, Ann Arbor, MI, United States; ^2^Louisiana State University Health Sciences Center, New Orleans, LA, United States; ^3^In Vivo Animal Core, University of Michigan Medical School, Ann Arbor, MI, United States; ^4^Unit for Laboratory Animal Medicine, University of Michigan Medical School, Ann Arbor, MI, United States; ^5^Department of Medicine, University of Pittsburgh, Pittsburgh, PA, United States; ^6^Critical Care Medicine Department, National Institutes of Health, Bethesda, MD, United States; ^7^Wayne State University School of Medicine, Detroit, MI, United States

**Keywords:** severe combined immunodeficiency rabbits, pneumonia, bacterial infection, *Pneumocystis*, Cas9 genome editing

## Abstract

Using the CRISPR/Cas9 gene-editing technology, we recently produced a number of rabbits with mutations in immune function genes, including FOXN1, PRKDC, RAG1, RAG2, and IL2RG. Seven founder knockout rabbits (F0) and three male IL2RG null (−/y) F1 animals demonstrated severe combined immunodeficiency (SCID), characterized by absence or pronounced hypoplasia of the thymus and splenic white pulp, and absence of immature and mature T and B-lymphocytes in peripheral blood. Complete blood count analysis showed severe leukopenia and lymphocytopenia accompanied by severe neutrophilia. Without prophylactic antibiotics, the SCID rabbits universally succumbed to lung infections following weaning. Pathology examination revealed severe heterophilic bronchopneumonia caused by *Bordetella bronchiseptica* in several animals, but a consistent feature of lung lesions in all animals was a severe interstitial pneumonia caused by *Pneumocystis oryctolagi*, as confirmed by histological examination and PCR analysis of *Pneumocystis* genes. The results of this study suggest that these SCID rabbits could serve as a useful model for human SCID to investigate the disease pathogenesis and the development of gene and drug therapies.

## Introduction

Severe combined immunodeficiency (SCID), caused by mutations in genes affecting lymphocyte development or function, such as *IL-2RG, RAG1* and/or *RAG2*, and *PRKDC*, accounts for the most severe phenotypes among primary immunodeficient patients (1, 2). SCID patients are highly susceptible to bacterial, viral, and fungal infections in early infancy, with the lungs being the most commonly affected site (1, 2). Mice have been the dominant animal species to model SCID. SCID mice produced by the conventional embryonic stem cell approach are widely used and commercially available (3, 4). Researchers also generated SCID mouse models using the emerging CRISPR/Cas9 nuclease tool (5, 6). However, their small body size and short lifespan sometimes limit their application in translational studies (7). Thus, development of a model utilizing an animal species with a larger body size and a longer lifespan is desirable to enhance the ability to predict clinical outcomes of the human SCID disease, especially given the fact that the SCID patients’ lifespan is expected to continue increasing.

In recent years, there has been increasing interest in using rabbit as a model for SCID because of its intermediate size, relatively long lifespan, and phylogenetic proximity to humans (8–10). Gene-editing nucleases especially CRISPR/Cas9 are efficient in generating double strand breaks (DSBs) in the target sequence in a wide spectrum of organisms including rabbits. These DSBs, if repaired by non-homologous end joining, often lead to frame-shift mutations that cause functional knockout (KO) of the gene (11). After establishing an efficient CRISPR/Cas9 based gene targeting platform in rabbits (12–14), we recently created multiple lines of SCID rabbits (8). Here, we report the clinical and pathologic findings in the SCID rabbits. This work presents a novel animal model to study human SCID.

## Materials and Methods

### Analysis of T and B Lymphocyte Development in the Peripheral Blood (PB) and Relevant Tissues

To analyze T- and B-lymphocytes in blood and relevant tissues, approximately 1 ml PB was collected from the rabbit central ear artery into a heparin-coated tube. Tissues from thymus, spleen, or bone marrow were separately harvested into a tissue culture dish and pressed with the plunger of a 3-ml syringe to isolate single cells. The single cells were transferred into a 50 ml tube with 10 ml of Flow cytometry staining buffer (PBS pH7.4, 0.1% BSA, 0.05% NaN3) and passed through a 70-µm cell strainer. The red blood cell lysis and antibody incubation were performed as described in our previous work (8). The suspended cells were tested by flow cytometry using MoFlo Astrios cell sorter (Beckman). The FACS data were analyzed using FlowJo software v10 (Tree Star, Ashland, OR, USA).

### Bacterial Identification by Bronchoalveolar Lavage Fluid (BALF) Culture

To identify bacteria in the rabbit lungs, we euthanized wild-type (WT) and SCID rabbits to lavage their lungs. The BALF of each animal was collected for bacterial culture. Briefly, the trachea was exposed, a small incision was made below the larynx. A sterilized three-way stopcock, connected with a sterile tubing, was inserted into the trachea *via* the incision and secured by a surgical thread, followed by flushing with sterile PBS three times to obtain the BALF. The BALF was inoculated to various bacterial culture plates including chocolate agar (Choc), anaerobic (ANA), tryptic soy agar (TSA), and Columbia colistin-nalidixic acid (CNA) plates, and incubated at 37°C for 48 h.

### Necropsy, Histology, and Histochemistry

A routine set of tissues were collected at necropsy following humane euthanasia, and immersion fixed in 10% neutral buffered formalin. Tissues were routinely trimmed and processed through graded alcohols, cleared with xylene, and embedded in paraffin. Tissues were sectioned at 5 μm, mounted on microscope slides, and stained with hematoxylin and eosin for microscopic analysis by a board-certified veterinary pathologist (Robert Sigler and Mark J. Hoenerhoff). To identify *Pneumocystis*, slides were stained with Gomori methenamine silver (GMS) stain. Briefly, slides were deparaffinized and rehydrated with distilled water, followed by heating with 2% chromic acid. Slides were rinsed in distilled water, then incubated in 1% sodium metabisulfite-methenamine silver solution in the presence of 0.5% Gold chloride, 5% Hypo, and working Light green. Following dehydration, slides were cleared and coverslipped.

### Complete Blood Count (CBC) Test

To assess hematologic parameters in blood of WT and SCID rabbits, at least 500 µl whole blood samples were drawn from rabbit central ear artery into an EDTA microtainer tube. Blood cells were counted using Sysmex XN-9100™ Automated Hematology System.

### Chest Radiographic Examination

Chest radiographic examination was performed on SCID rabbits under sedation with dexmedetomidine (0.2 mg/kg intramuscular injection) using SEDECAL APR-VET Digital X-ray Systems. Atipamezole (same volume as dexmedetomidine) was intramuscularly injected to aid in recovery of animals.

### Estimation of the 16S/18S rRNA Ratio in the Lungs of SCID Rabbits

To measure the abundance of bacterial 16S rRNA genes, the ratio of 16S to 18S rRNA genes was calculated based on copy number determined by quantitative PCR (q-PCR). Total DNAs were extracted from lung samples using DNeasy Blood & Tissue Kits (Qiagen). q-PCR was performed using iQ SYBR Green Supermix (Bio-Rad). The 16S abundance was normalized to 18S. The primers used to amplify the 16S and 18S rRNA genes are as follows:

16S-F1, 5′-AGTCGACAGGAGGCAGCAGTRRGGAAT-3′;
16S-R1, 5′-AGTCGACACTACCRGGGTATCTAATCC-3′;
18S-F1, 5′-GTTGGTGGAGCGATTTGTCTG-3′;
18S-R1, 5′-GGCTGAACGCCACTTGTCC-3′.

### Statistical Analysis

Data are presented as either mean ± SEM or individual data points. Statistically significant differences between genotype groups were determined using either the Student’s *t*-test or one-way ANOVA and the Student–Newman–Keuls test. Differences were considered statistically significant if *P* < 0.05.

## Results

### SCID Rabbits Fail to Thrive and Survive

We generated a variety of different KO rabbits with a SCID phenotype by using CRISPR/Cas9-mediated gene targeting (8). As a result of the extremely high KO efficiency of the CRISPR/Cas9 system, seven of 17 (41.2%) founder (F0) KO animals developed the SCID phenotype. Of these, four were single-gene-knockout in *Il2rg* (*n* = 3, ID#: 182, 186, and 194) or *Prkdc* (*n* = 1, ID#: 191), one was double-gene KO in *Rag1* and *Rag2* (ID#: 196), one was triple-gene KO in *Foxn1, Prkdc*, and *Rag1* (ID#: 179), and one was quadruple-gene KO in *Foxn1, Prkdc, Rag1*, and *Il2rg* (ID#: 181). Due to the nature of CRISPR/Cas9-mediated gene disruption, the F0 generation SCID rabbits are typically mosaic and carry more than one type of mutation. Detailed genotyping information of these animals is shown in supplementary data (Table S1 in Supplementary Material).

**Figure 1:**
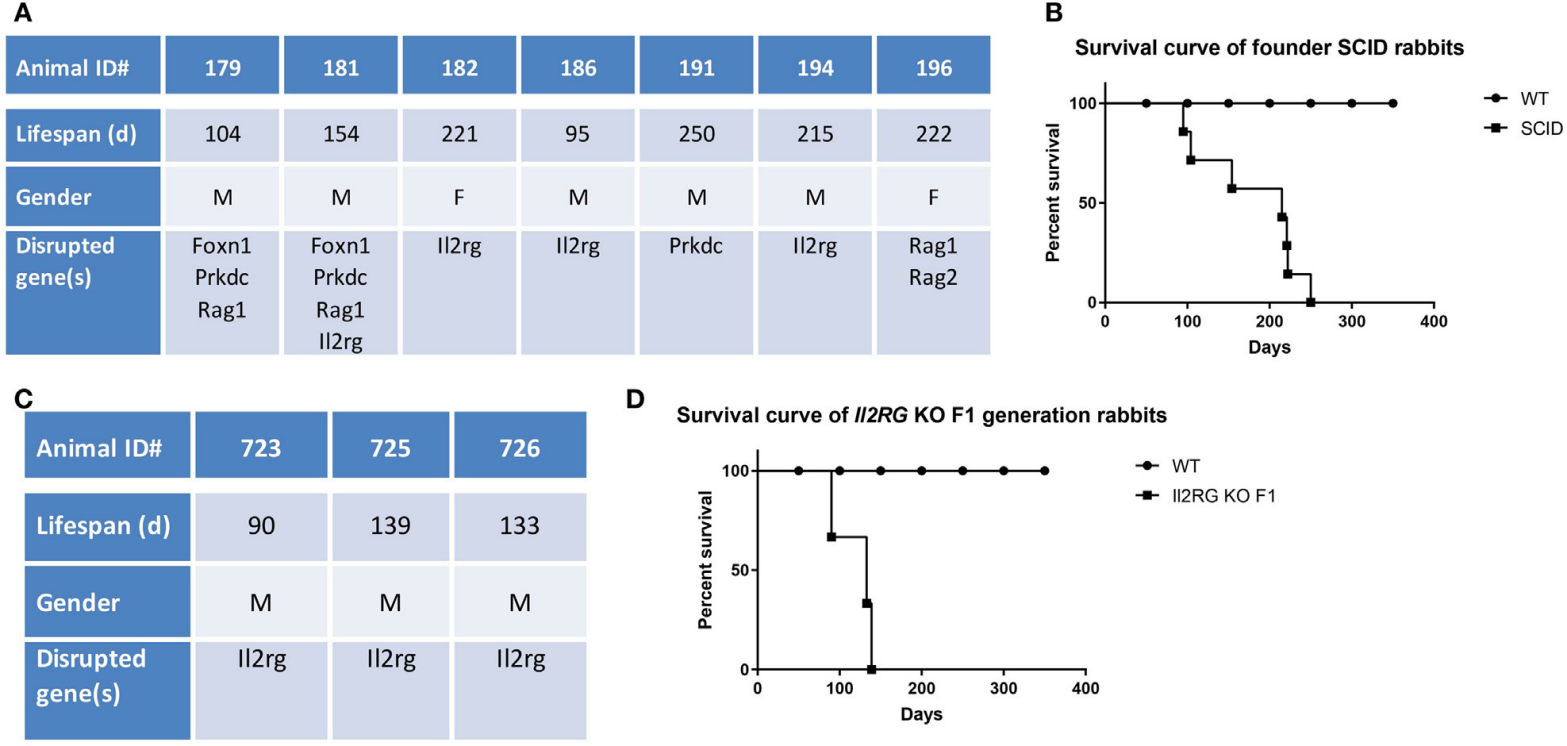
Lifespan of severe combined immunodeficiency (SCID) rabbits. **(A)** Summary of founder (F0) SCID rabbits. **(B)** Survival curve of Founder SCID rabbits. **(C)** Summary of F1 IL2RG-KO (X-SCID) rabbits. **(D)** Survival curve of F1 IL2RG-KO (X-SCID) rabbits.

The median lifespan of the SCID founder rabbits (*n* = 7) was 215 days, ranging from 95 to 250 days (Figures 1A,B). By 250 days, all these seven founder SCID rabbits had died or had been euthanized due to morbidity, whereas the WT immunocompetent control rabbits (*n* = 10) had a 100% survival rate.

Through conventional breeding, we subsequently obtained three X-SCID (*Il2rg^−/y^*) male rabbits in the F1 generation (ID#: 723, 725, and 726) by breeding *IL2rg*-KO founders with WT rabbits. Genotypes of these three animals are shown in supplementary data (Table S1 in Supplementary Material).

The median lifespan of these F1 X-SCID rabbits (*n* = 3) was 133 days, ranging from 90 to 139 days (Figures 1C,D), significantly shorter than those of the founder SCID rabbits (*P* < 0.05), possibly due to their complete KO status compared to the mosaic background of the founder SCID rabbits.

### SCID Rabbits Are Depleted of Functional B- and T-Lymphocytes

**Figure 2:**
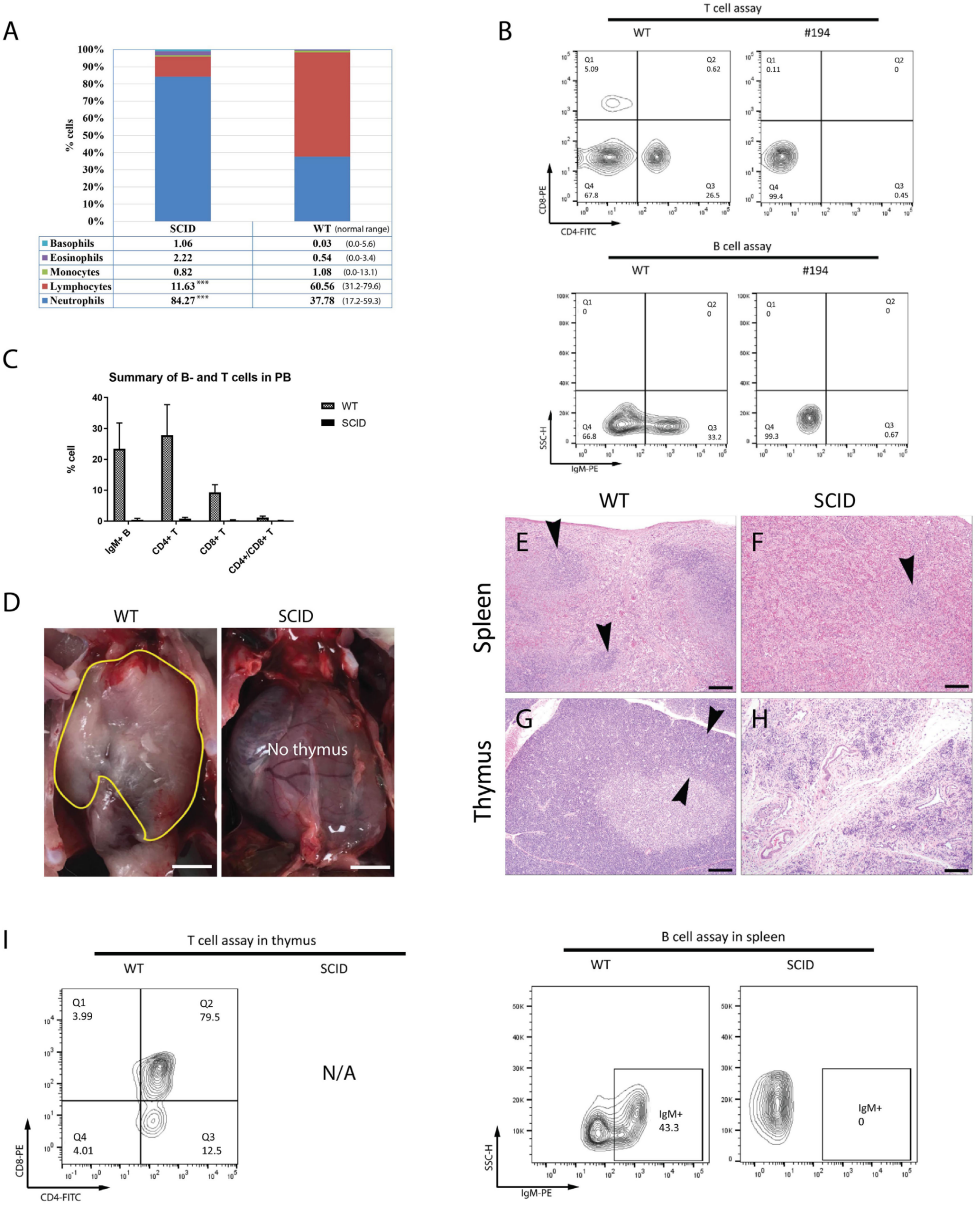

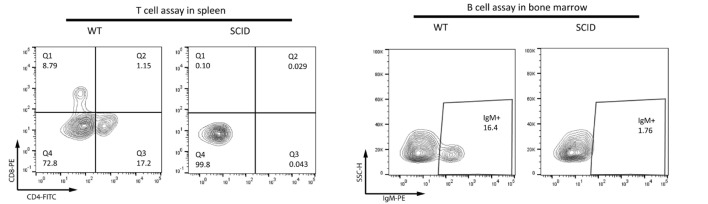
Founder severe combined immunodeficiency (SCID) rabbits have combined immunodeficiency. **(A)** Summary of complete blood count (CBC) results. **(B)** Representative flow cytometry results of SCID rabbit. **(C)** Summary of flow cytometry results of B- and T-cells in peripheral blood (PB) in all founder SCID rabbits. **(D)** Thymus in SCID and wild-type (WT) rabbits. **(E,F)** Histology of spleen and thymus in SCID founder rabbit. In contrast to WT animals **(E)**, spleens from knockout (KO) animals **(F)** were almost completely devoid of lymphocytes, save for a few small clusters of cells (arrowhead). Similarly, the thymus in WT animals **(G)** had normal cortical thickness (arrowheads) and dense cortical lymphocytes, while thymus from KO animals **(H)** had complete loss of the thymic cortical structure with almost no lymphocytes present, within a background of thymocytes and supporting stroma. **(I)** Flow cytometry results of B- and T-cells harvested from spleen and bone marrow. Representative individual results and images are from animal #194 unless otherwise indicated.

Complete blood count analysis revealed that the SCID founder rabbits (#179, 181, 182, 194, and 196) were severely leukopenic and lymphocytopenic, characterized by extremely low white blood cell (WBC 1.90–3.32 K/µl vs. 5.2–12.5 K/µl normal range) and lymphocyte (LY 0.20–0.64 K/µl vs. 1.6–10 K/µl normal range) counts. Percentage wise, SCID founder rabbits had low lymphocytes and high heterophils (rabbit neutrophils) compositions in the PB, a reversed pattern as compared to WT rabbits (Figure 2A), consistent with the SCID phenotype and suggesting a systemic infection.

In the PB, populations of IgM+ B cells, CD4+ T cells, CD8+ T cells, and CD4+/CD8+ T cells of SCID founder rabbits (#179, 181, 182, 186, 190, 194, 196) were analyzed by flow cytometry (Figures 2B,C; Figures S1 and S2 in Supplementary Material). As expected, all SCID founder rabbits had only minimal numbers of these immune cells, significantly lower compared to the WT controls (*P* < 0.001). Similar findings were observed on flow cytometry analysis of F1 animals (#723, 725, 726; Figures S3 and S4 in Supplementary Material).

Postmortem examination revealed that the thymus was either markedly reduced in size or completely absent in SCID rabbits (Figure 2D). Compared to WT animals, in which cortical and medullary zones were observed in appropriate thickness, and there were adequate numbers of cortical lymphocytes present (Figure 2G), the SCID animals exhibited marked depletion of all lymphoid elements, with only remnants of medullary thymocytes and sparse, scattered mononuclear cells and granulocytes present in the thymus (Figure 2H). Similarly, compared to WT rabbits whose spleen had clear zones of white and red pulp (Figure 2E), the SCID rabbits were markedly depleted of all lymphoid elements in the spleen (Figure 2F).

Consistent with the histological findings, flow cytometry analysis showed that, in contrast to the WT animals, IgM+ B cells in the spleens and bone marrow as well as CD4+ and CD8+ T cells in the thymus (where available) and spleen were very scarce and significantly lower in SCID rabbits (*P* < 0.001) (Figure 2I).

The lack of functional B- and T-lymphocytes in these SCID rabbits is consistent with a typical phenotype of combined immunodeficiency. Due to the lack of working antibodies to rabbit natural killer (NK) cells, we were not able to characterize NK cell profiles in these animals.

### SCID Rabbits Are Prone to Bacterial and *Pneumocystis* Pulmonary Infections

**Figure 3:**
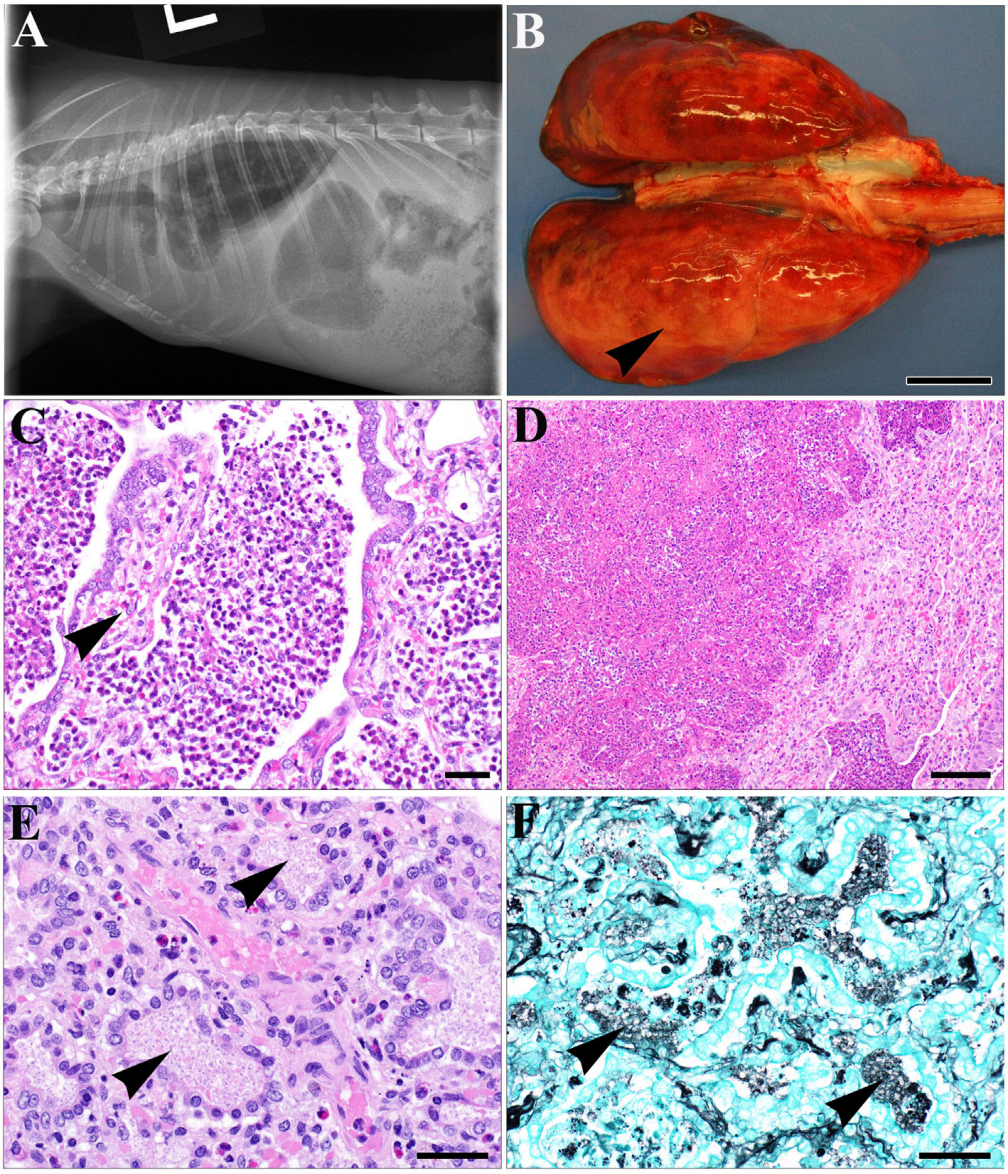
Gross pathology and histopathology of knockout rabbits with pneumonia. (**A)** Left lateral thoracic radiograph of a severe combined immunodeficiency rabbit (#725) with pleural effusion. A soft tissue opacity was observed in both the caudal and ventral thoracic cavity, obscuring the cardiac silhouette and causing dorsal retraction of the lung fields. The radiographic appearance is consistent with pleural effusion. **(B)** Lungs of affected animals were diffusely expanded, variably mottled dark red to tan, often containing lateral rib impressions (arrowhead). Bar = 1 cm. **(C)** Marked inflammatory cell infiltrate centered on bronchioles and alveoli composed of heterophils, fewer macrophages and cellular debris, with denudation of bronchiolar epithelium and partial filling of bronchiolar lumens with granulation tissue (arrowhead). Bar = 20 μm. **(D)** Multifocal to coalescing zones of alveolar wall necrosis and filling with degenerate heterophils and cellular debris, with atelectasis of adjacent pulmonary parenchyma. Bar = 50 μm. **(E)** Alveolar spaces are lined by type II pneumocyte hyperplasia and filled with faintly eosinophilic, foamy material (arrowheads). Bar = 20 μm. **(F)** Gomori methenamine silver staining of lung tissue demonstrating clusters of *Pneumocystis* cysts (arrowheads) within alveolar spaces. Bar = 20 μm. Representative individual results and images are from animal #196 unless otherwise indicated.

Without prophylactic antibiotics these SCID rabbits developed symptoms and signs of respiratory tract infection and ultimately succumbed to respiratory failure. Clinically, animals exhibited labored breathing, tachypnea, anorexia, and lethargy, and one presented with pleural effusion on radiographs (Figure 3A). On gross necropsy examination, lungs from the SCID animals were variably mottled dark red to tan, often with pinpoint white foci diffusely scattered along the pleural surface. Some animals presented with dark red, consolidated cranial lung lobes, which were firm on palpation and sank in fixative. Overall, there was diffuse expansion of the dorsal lung lobes, with the presence of rib impressions in some animals (Figure 3B). Histologically, lesions in most animals resembled an interstitial pneumonia, with or without concurrent bronchopneumonia. Bronchopneumonia was characterized by variably severe inflammatory cell infiltrates composed predominantly of heterophils, centered primarily on airways. Within airways, there were multifocal accumulations of heterophils admixed with fewer macrophages, fibrin, and cellular debris (Figure 3C), which were often associated with erosion of bronchiolar epithelium and replacement by exuberant granulation tissue. The inflammatory infiltrates often extended into the deeper alveolar spaces, with associated pulmonary edema, fibrin deposition, and multifocal alveolar wall necrosis (Figure 3D). Interstitial pneumonia was characterized by consolidation of the pulmonary parenchyma due to thickening of the alveolar walls with cellular infiltrates, multifocal interstitial fibrosis, and proliferation of type II pneumocytes, with the presence of foamy faintly eosinophilic material (Figure 3E).

**Figure 4:**
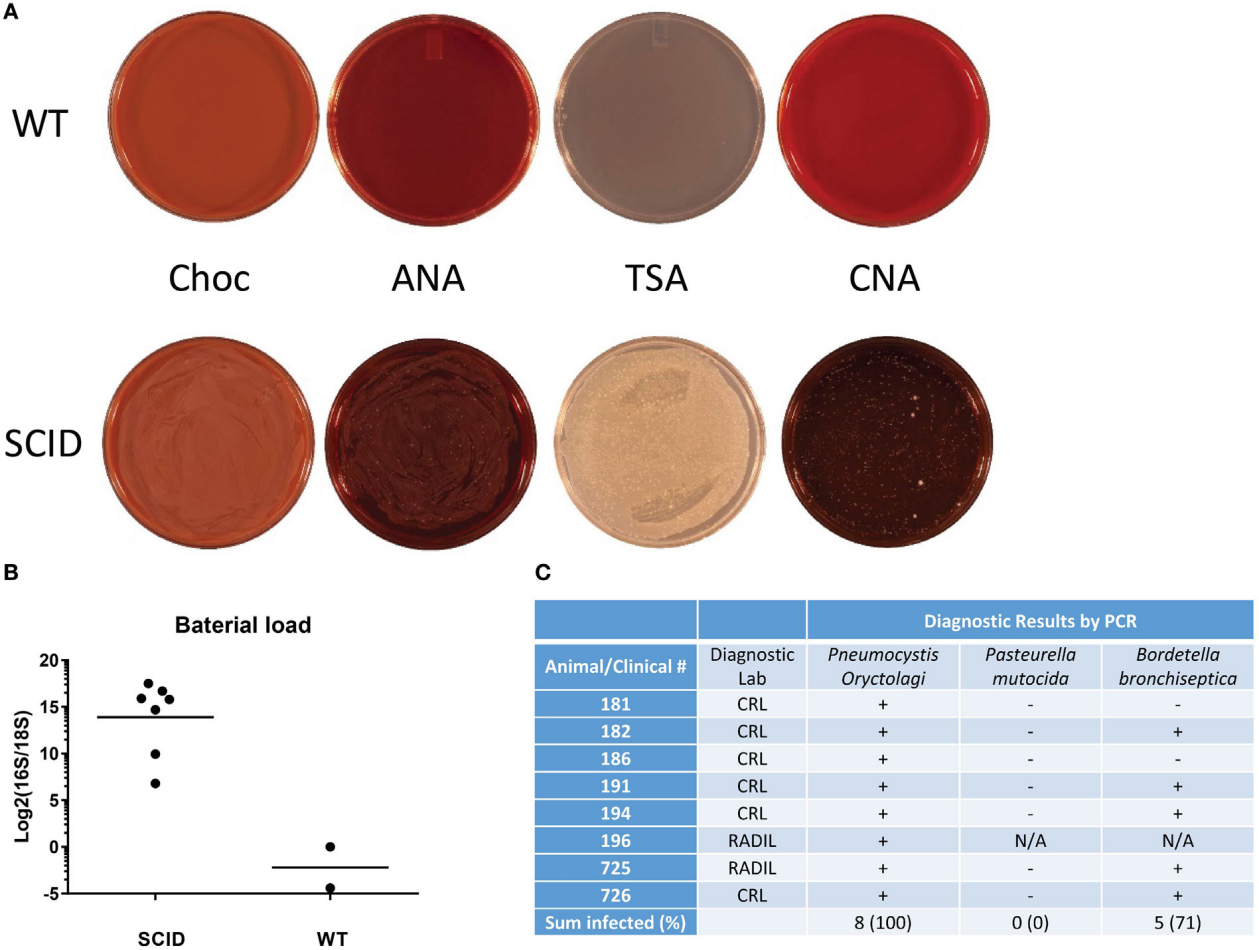
Bacterial and *P. oryctolagi* identification in severe combined immunodeficiency (SCID) rabbits. **(A)** Bronchoalveolar lavage culture of SCID and wild-type (WT) rabbits on chocolate (Choc), anaerobic (ANA), tryptic soy agar (TSA) and Columbia colistin-nalidixic acid (CNA) plates. **(B)** Bacterial load, indicated by 16S/18S rRNA gene ratio, in the lungs of SCID and WT rabbits. **(C)** Diagnostic PCR results for *P. oryctolagi, P. mutocida*, and *B. bronchiseptica* in the lungs of SCID rabbits.

Due to severely compromised adaptive immune system in the SCID rabbits, we wanted to identify the common pathogens that infected the lungs of SCID rabbits. Bronchoalveolar lavage (BAL) fluids from moribund animals that were euthanized were immediately collected and sent to clinical diagnostic laboratories for bacterial culture and identification. The results showed that *Bordetella bronchiseptica* was present in most SCID rabbits (5/8, 71%). *Pasteurella mutocida*, another common rabbit respiratory pathogen, was not observed in any BAL culture (Figure 4C).

We measured the ratio of 16S/18S ribosome RNA genes in the total DNA extract from the lungs of SCID rabbits in comparison with WT rabbits (Figure 4B). The ratio was extremely low in the WT rabbits, indicating absence of bacterial infections. In SCID rabbits, the ratios were significantly elevated (*P* < 0.001), ranging from 109- to 182,803-fold increase, suggesting severe bacterial loads in the lungs of SCID rabbits, consistent with the observation of bacterial growth in the culture of BAL from the SCID but not WT rabbits (Figure 4A).

Since *Pneumocystis* is a major cause of pneumonia in both primary and acquired immunodeficient patients, we investigated if *Pneumocystis oryctolagi (P. oryctolagi)*, the rabbit-specific *Pneumocystis* species (15), was present in post-weaning SCID rabbits. Immunocompetent rabbits would harbor this organism at low levels until shortly after weaning, at which time they mount a cell-mediated immune response and clear the organism. In post-weaning SCID rabbits, however, severe *P. oryctolagi* infection was observed. The lung alveoli were filled with abundant acellular eosinophilic foamy exudate in hematoxylin–eosin staining (Figure 3E), which is characteristic of *Pneumocystis* pneumonia (16). Clusters of *Pneumocystis* organisms in the alveoli were confirmed with GMS staining (Figure 3F). PCR tests by clinical diagnostic laboratories revealed that all SCID rabbit lungs were positive for *P. oryctolagi* sequences (Figure 4C).

## Discussion

Respiratory complications are a major cause of morbidity and mortality in human SCID patients. Infants with typical SCID come to medical attention between ages 3 and 6 months with failure to thrive, oral/diaper candidiasis, absent tonsils and lymph nodes, and recurrent viral, bacterial, and fungal infections. Without intervention, SCID patients usually acquire severe infection and die by age 2 years (1, 17). The SCID rabbits in the present study sustained well until weaning, at which time the animals began to deteriorate due to respiratory illness caused by opportunistic infections with *Pneumocystis* and *B. bronchiseptica*. This course of disease onset and development closely mimics that of human SCID; therefore, SCID rabbits represent new translational tools to the research community with particular relevance to lung infection phenotypes. In addition to lung pathologies, these animals also suffer from infections in other organs such as kidney and liver, which is being further investigated and beyond the scope of this report.

*Pneumocystis* is a ubiquitous opportunistic fungal pathogen of mammals with strict host-species specificity. The species infecting humans, *P. jirovecii*, can cause asymptomatic or mild infection in immunocompetent persons but life-threatening pneumonia (*Pneumocystis* pneumonia or PCP) in patients with primary immunodeficiency and acquired immunodeficiency (16). Rodent models have made invaluable contributions toward our understanding of the biology, therapy, and host responses to *Pneumocystis* infection that are relevant to human disease (18). Although less commonly used than rodents, studies of rabbits infected by *P. oryctolagi* have revealed some distinct features. Compared to rodents, healthy rabbits can develop relatively heavy *Pneumocystis* infection either spontaneously at weaning or following cohousing with infected rabbits (19, 20). This spontaneous, natural infection is usually resolved within 3–4 weeks. Hence, normal rabbits (without immunosuppression) have been used to study the pathogen–host interaction, particularly the host immune responses, during natural *Pneumocystis* infection in normal hosts (21–23). Another distinct finding from the rabbit PCP model is that *P. oryctolagi* organisms are usually detached from each other, lining along the alveolar epithelium, in contrast to rodent *Pneumocystis* which are usually clustered together, filling the alveolar lumen (15). Other striking features of *P. oryctolagi* include a shorter doubling time and a higher ratio of cyst/trophic form in infected rabbit lungs (15) compared to rodent *Pneumocystis*. These features raise a variety of questions about the underlying forces shaping the *P. oryctolagi* species. A detailed understanding of these features and their underlying mechanisms may provide new insights into the biology, pathogenesis, and intervention of *Pneumocystis* relevant to human disease.

All rabbits examined appear to be co-infected by common primary lung bacterial pathogens (based on the 16S/18S data) and *P. oryctolagi* in the present work. The results are within our expectation as no prophylactic antibiotics were used to these animals and co-infection of bacterial and *Pneumocystis* pathogens is not rare in immunodeficient patients (24). It remains to be tested whether increased barrier facilities and administration of bacterial specific antibiotics are able to establish *P. oryctolagi* only/dominant infections in SCID rabbits.

In addition to the unique features of rabbit PCP, SCID rabbits present several other advantages. First, based on phylogenetic analysis of the genes that determine antibiotic sensitivity to trimethoprim and sulfamethoxazole, rabbit *P. oryctolagi* is more closely related to human *P. jirovecii*, than rodent derived species (25). It is possible that information gained from studies of rabbit models may be more applicable to human disease than are the rodent models.

Second, rabbits are of relatively large size. Adult NZW rabbits weigh 3–5 kg, comparable to those of human infants, which would help facilitate translation of imaging diagnostics and clinical modalities developed in SCID rabbits relatively adaptable for infant patients (26). Testing of intervention procedures, such as I.V. infusion, will be more easily established in SCID rabbits, thanks to the relatively large diameter and easy access to the ear veins. Some translational studies that currently cannot be easily tested in rodents due to their small size, for example, bone marrow aspiration and autologous hematopoietic cell transplantation after *ex vivo* gene correction (27, 28), will be more feasible using SCID rabbits.

Furthermore, the laboratory rabbit has an expected lifespan of 6 years or beyond, allowing for more clinically relevant long-term assessment of potential therapeutics (29). All SCID rabbits used in the present work were housed under normal SPF facility conditions with immunocompetent peers, exposing them to commensal pathogens, and prophylactic antibiotics were not instituted. Therefore, these rabbits eventually developed opportunistic infections and died at an early age. It is expected that by housing these animals under maximum-barrier conditions with enhanced sanitary practice will greatly reduce the chances of lung infections, therefore, increasing the lifespan of these animals. Indeed, currently SCID rabbits housed under positive pressure HEPA-filtered flexible film isolator biobubble conditions and treated with prophylactic antibiotics have reached 10 months of age without signs of disease (data not shown). It is anticipated that SCID rabbits will outlive their mouse counterparts, therefore, providing an animal model for long-term studies.

Compared to other large animals such as pigs, dogs, and monkeys, rabbits have a shorter gestation term (30–31 days), lower maintenance cost, and easy adaptability to common research facilities.

It is also noteworthy the rabbit is a classic animal model for the study of some infectious diseases and particularly has long been considered a preferred model over mice for the study of *Staphylococcus aureus* infections in the lungs, a major threat to SCID patients, which is not modeled well in rodents (30–32).

*In summary*, we have established SCID rabbit models, which developed bacterial and *Pneumocystis* pulmonary infections. These rabbit models of SCID may be useful for the study of these devastating diseases secondary to primary immunodeficiency syndromes, with the potential to facilitate development of early diagnostics and therapeutics for immunodeficient patients.

## Ethics Statement

The animal maintenance, care and use procedures were reviewed and approved by the Institutional Animal Care and Use Committee of the University of Michigan, an AAALAC International accredited facility. All procedures were carried out in accordance with the approved guidelines. The immunodeficient rabbits were housed in a standard specific pathogen-free facility. All rabbits including SCID rabbits were monitored daily, and moribund rabbits were euthanized and necropsied. For breeding the immunodeficient rabbits, WT NZW rabbits were obtained from Covance or Charles River Laboratories.

## Author Contributions

JX conceived the idea. JX, YEC and FS designed the experiments. JS, GW, MH, JR, DY, JZ, JY, PL, RS, MB, SE, KEC, KOC, JK, LP, and LM conducted experiments. JS, GW, MH, FS, LM, and JX wrote and revised the manuscript.

## Conflict of Interest Statement

The authors declare that the research was conducted in the absence of any commercial or financial relationships that could be construed as a potential conflict of interest.
