# Supplementary material for: Bacterial and Pneumocystis Infections in the Lungs of Gene-Knockout Rabbits with Severe Combined Immunodeficiency
Source: Front Immunol. 2018 Mar 9;9:429. doi: 10.3389/fimmu.2018.00429 (PMC5854650; doi:10.3389/fimmu.2018.00429)
Supplement: Supplementary file 1 [file table_1.DOCX]

**Supp. Table 1. Genotypes of SCID rabbits.** Underlined: sgRNA targeting sequences. Blue letters: Protospacer adjacent motif (PAM) sequences.

| Animal ID# | Mutant gene(s) | Mutant sequence/WT sequence |
| --- | --- | --- |
| Founders (F0) |  |  |
| 179 | Foxn1 | CGGGGCCCGGTGACGCAATGCTGGGGC-----------AGGGAGGGTGCC (-11)  CGGGGCCCGGTGACGCAATGCTGGGGCTGTGCGG**GGG**CAGGGAGGGTGCC (WT) |
|  | Rag1 | GGAGAAGAATTCCTCTGAGGGGAAGC------TTGAGCAATCTCCAGCAG (-6)  GGAGAAGAATTCCTCTGAGGGGAAG**CCC**TCCCTTGAGCAATCTCCAGCAG (WT) |
|  | Prkdc | GCTGGCAGCCGGCCAC-GCTGCGCCGCAGCGGTCCCCGCACGACAAGAAA (-1)  GCTGGCAGCCGGCCACAGCTGCG**CCG**CAGCGGTCCCCGCACGACAAGAAA (WT) |
| 181 | Foxn1 | CGGGGCCCGGTGACGCAATGCTGGGGCTG-------------AGGGTGCC (-13)  CGGGGCCCGGTGACGCAATGCTGGGGCTGTGCGG**GGG**CAGGGAGGGTGCC (WT) |
|  | Rag1 | GGAGAAGAATTCCTCTGAGGGGAAGC------------AATCTCCAGCAG (-12)  GGAGAAGAATTCCTCTGAGGGGAAG**CCC**TCCCTTGAGCAATCTCCAGCAG (WT) |
|  | Il2rg | CATTTTACCCTTTGAAGTTTTGCTCCCCCAAAAAGATCGGGTGGCTCCATTCACTCCAATGCTGAG (+4-3)  CATTTTACCCTTTGAAGTTTTGCT**CCC**CCAGTGGATCGGGTGGCTCCATTCACTCCAATGCTGAG (WT) |
|  | Prkdc | GCTGGCAGCCGGCCACAGCTGCGCCGCAGCGCGTCCCCGCACGACAAGAAA (+1)  GCTGGCAGCCGGCCACAGCTGCG**CCG**CAGCGGTCCCCGCACGACAAGAAA (WT) |
| 182 | Il2rg | CATTTTACCCTTTGAAGTTTTGCTCCCCCA-TGGATCGGGTGGCTCCATTCACTCCAATGCTGAG (-1)  CATTTTACCCTTTGAAGTTTTGCT**CCC**CCAGTGGATCGGGTGGCTCCATTCACTCCAATGCTGAG (WT) |
| 186 | Il2rg | CATTTTA--------------GCTCCCCCAGTGGATCGGGTGGCTCCATTCACTCCAATGCTGAG (-14)  CATTTTACCCTTTGAAGTTTTGCT**CCC**CCAGTGGATCGGGTGGCTCCATTCACTCCAATGCTGAG (WT) |
| 191 | Prkdc | GCTGGCAGCCGGC-------------------------CACGACAAGAAA (-25)  GCTGGCAGCCGGCCACAGCTGCG**CCG**CAGCGGTCCCCGCACGACAAGAAA (WT) |
| 194 | Il2rg | CATTTTACCCTTTGAAGTTT---------------------GGCTCCATTCACTCCAATGCTGAG (-21)  CATTTTACCCTTTGAAGTTTTGCT**CCC**CCAGTGGATCGGGTGGCTCCATTCACTCCAATGCTGAG (WT) |
| 196 | Rag1 | GGAGAAGAATTCCTCTGAGGGGAAGCCCTCAGGAGCAATCTCCTTGAGCAATCTCCAGCAG (+11bp)  GGAGAAGAATTCCTCTGAGGGGAAG**CCC**TCCCTTGAGCAATCTCCAGCAG (WT) |
|  | Rag2 | AGGATTCCTGCTATCTTCCTCCTCTGCACTGAAAGGAACGTTACCCAGCCACTTGCACATTC (-1+13)  AGGATTCCTGCTATCTT**CCT**CCTCTCCGTTACCCAGCCACTTGCACATTC (WT) |
| F1 animals |  |  |
| 723 | Il2rg | CATTTTACCCTTTGAAGTTTTGCTCCCCCA-TGGATCGGGTGGCTCCATTCACTCCAATGCTGAG (-1bp)  CATTTTACCCTTTGAAGTTTTGCT**CCC**CCAGTGGATCGGGTGGCTCCATTCACTCCAATGCTGAG (WT) |
| 725 | Il2rg | CATTTTACCCTTTGAAGTTTTGCTCCCCCA-TGGATCGGGTGGCTCCATTCACTCCAATGCTGAG (-1bp)  CATTTTACCCTTTGAAGTTTTGCT**CCC**CCAGTGGATCGGGTGGCTCCATTCACTCCAATGCTGAG (WT) |
| 726 | Il2rg | CATTTTACCCTTTGAAGTTTTGCTCCCCCA-TGGATCGGGTGGCTCCATTCACTCCAATGCTGAG (-1bp)  CATTTTACCCTTTGAAGTTTTGCT**CCC**CCAGTGGATCGGGTGGCTCCATTCACTCCAATGCTGAG (WT) |
