# Supplementary figures and images for: Bacterial and Pneumocystis Infections in the Lungs of Gene-Knockout Rabbits with Severe Combined Immunodeficiency
Source: Front Immunol. 2018 Mar 9;9:429. doi: 10.3389/fimmu.2018.00429 (PMC5854650; doi:10.3389/fimmu.2018.00429)

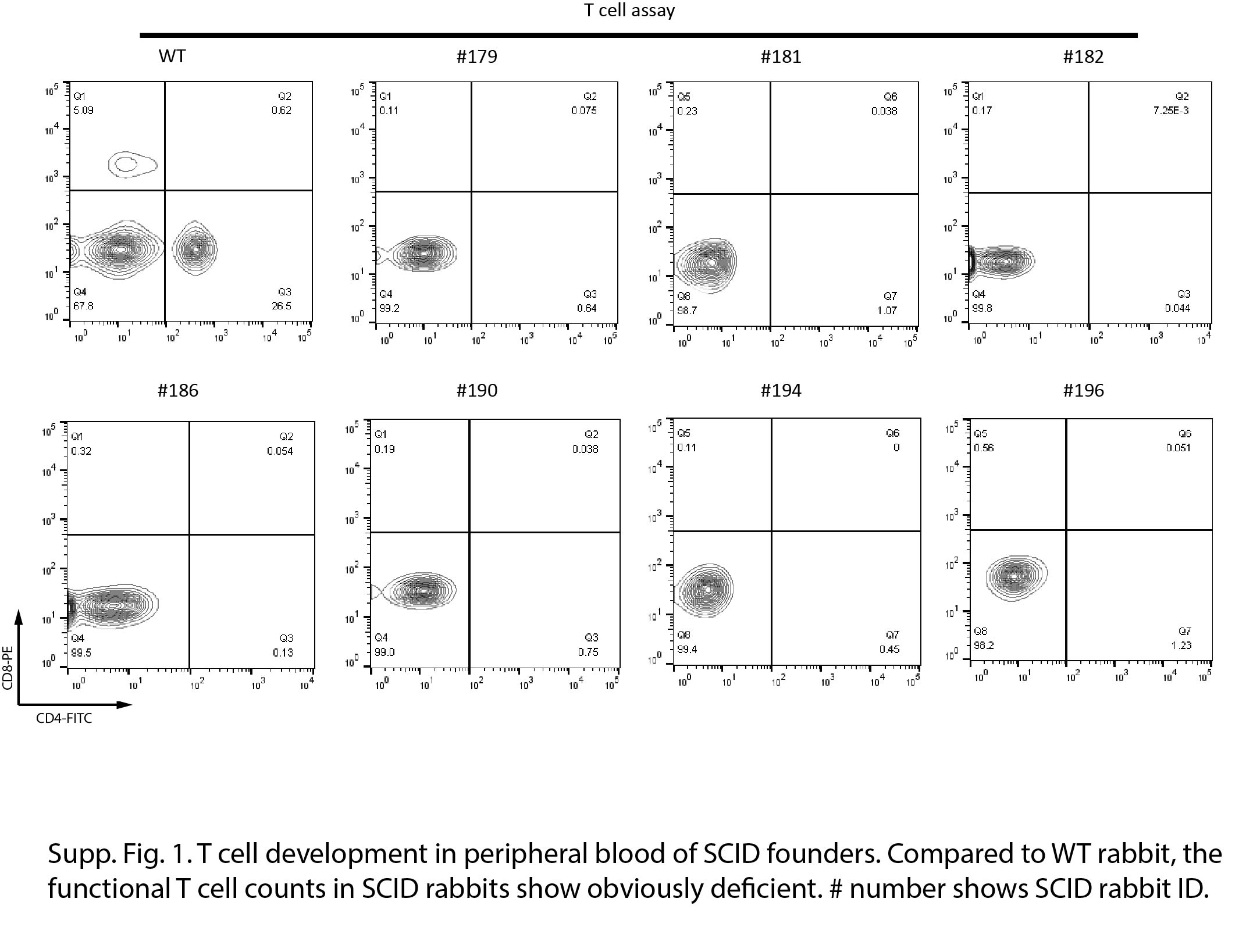

Supplement: Supplementary file 2 [file image_1.JPEG]

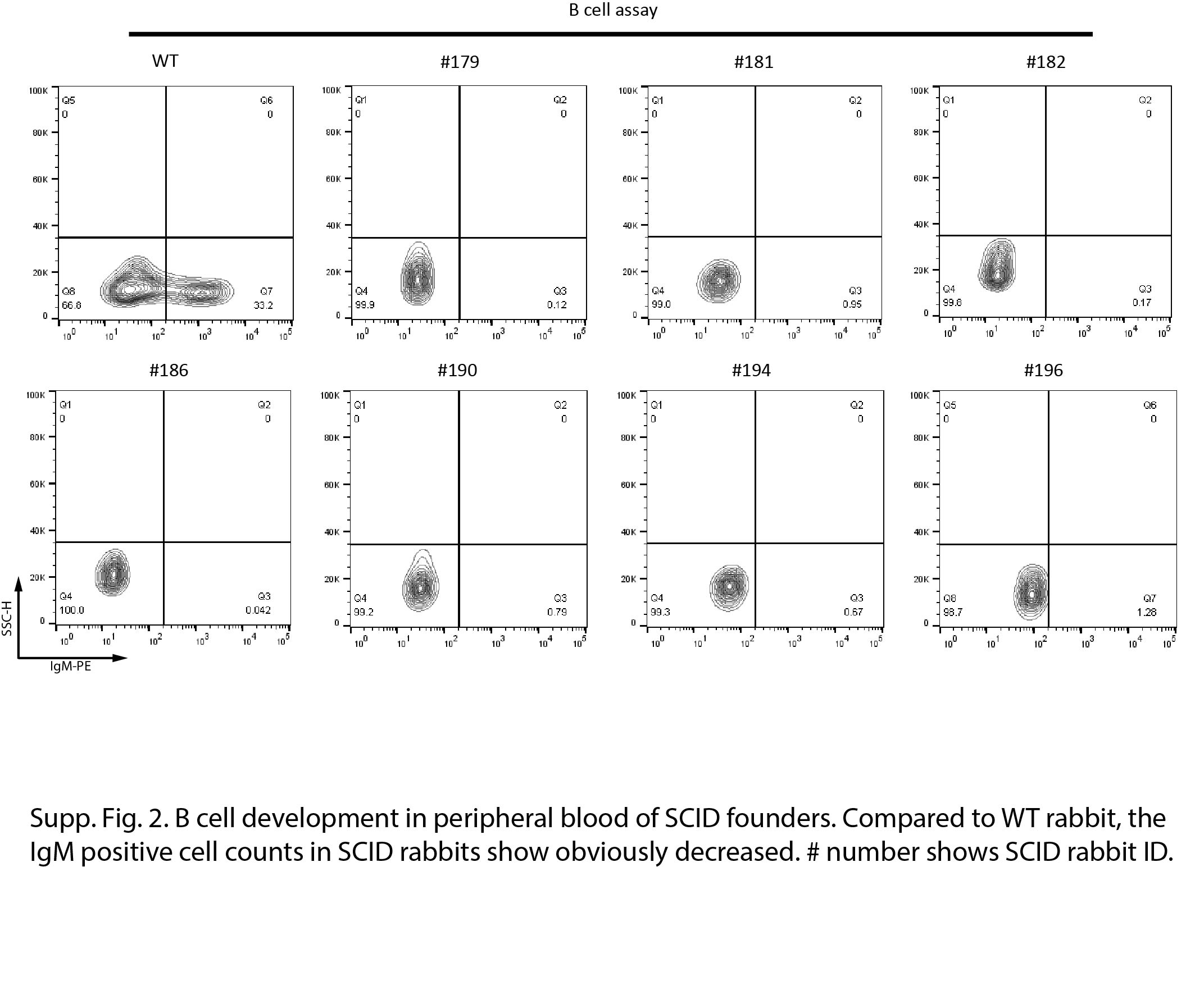

Supplement: Supplementary file 3 [file image_2.JPEG]

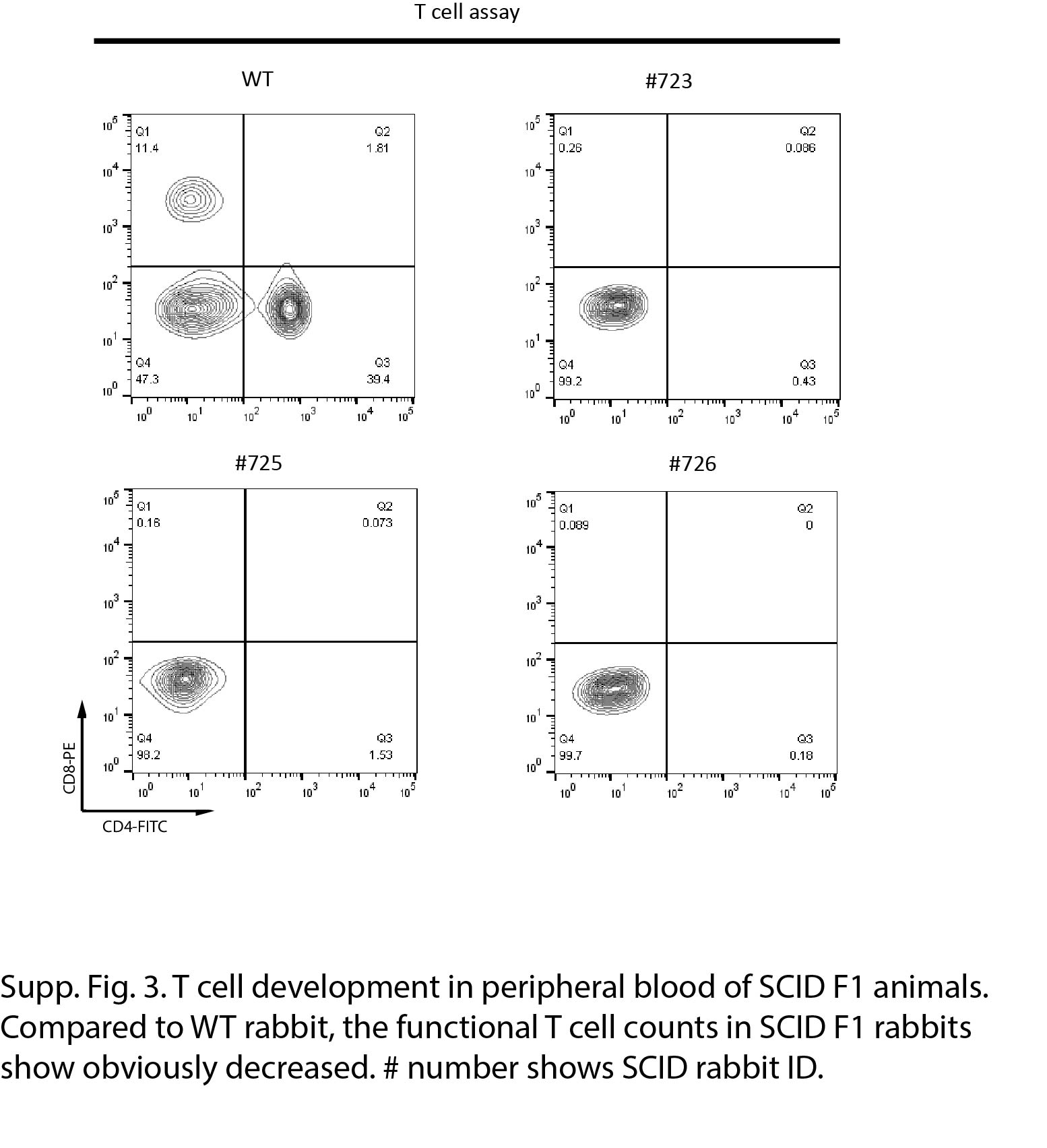

Supplement: Supplementary file 4 [file image_3.JPEG]

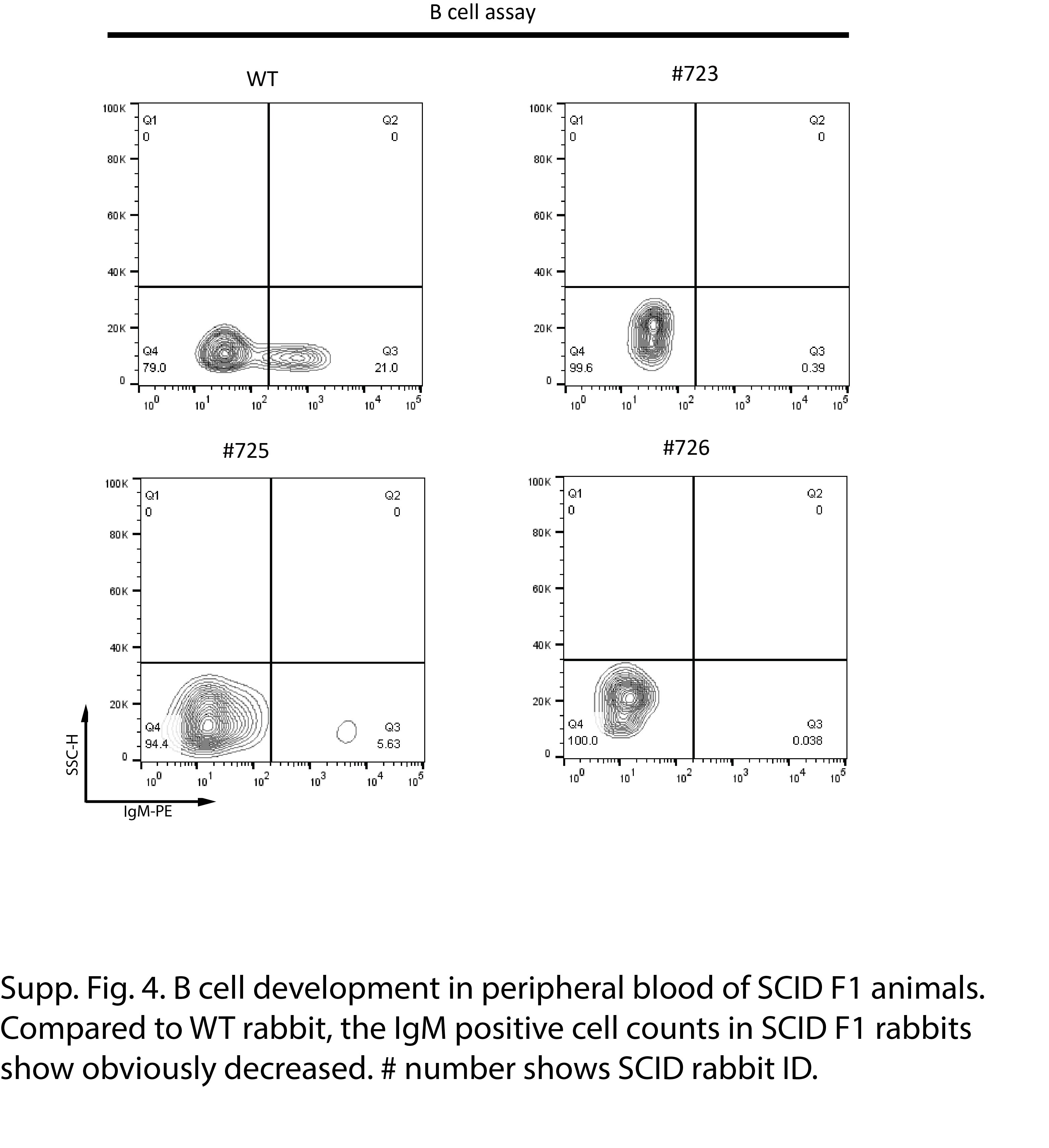

Supplement: Supplementary file 5 [file image_4.JPG]
